# Drain fluid and serum amylase concentration ratio is the most reliable indicator for predicting postoperative pancreatic fistula after distal pancreatectomy

**DOI:** 10.1186/s12893-023-01980-1

**Published:** 2023-04-12

**Authors:** Masahiro Fukada, Katsutoshi Murase, Toshiya Higashi, Itaru Yasufuku, Yuta Sato, Jesse Yu Tajima, Shigeru Kiyama, Yoshihiro Tanaka, Naoki Okumura, Takao Takahashi, Nobuhisa Matsuhashi

**Affiliations:** Department of Gastroenterological Surgery, Gifu University Hospital, 1-1 Yanagido, Gifu City, Gifu, 501-1194 Japan

**Keywords:** Drain fluid and serum amylase concentration ratio, Postoperative pancreatic fistula, Distal pancreatectomy

## Abstract

**Background:**

Postoperative pancreatic fistula (POPF) is a major complication of pancreatic surgery. Drain fluid amylase concentration (DAC) is considered a predictive indicator of POPF. However, other indicators related to postoperative drain fluid amylase status exist, and the most reliable indicator for predicting POPF remains unclear. The object of this study is to identify the single most accurate indicator related to drain fluid amylase status of POPF after distal pancreatectomy (DP).

**Methods:**

This single-institution retrospective study included 122 patients who underwent DP. The study was conducted between 2010 and 2022 at Gifu University Hospital. We statistically analyzed DAC, drain fluid amylase amount (DAA) calculated by multiplying DAC and daily drainage volume, and drain and serum amylase concentration ratio (DSACR) to assess the correlation with POPF.

**Results:**

Based on the definition and grading of the International Study Group of Pancreatic Fistula, 24.6 (%) of the 122 patients had Grades B and C POPF. The result of the receiver operating characteristic (ROC) curve for predicting POPF after DP, DSACR had the highest area under curve(AUC) value among DAC, DAA, and DSACR both POD1 and POD3. The cutoff value of DSACR on POD1 was 17 (AUC 0.69, sensitivity 80.0%, specificity 58.2%, and accuracy 63.6%). The cutoff value of DSACR on POD3 was 22 (AUC 0.77, sensitivity 77.7%, specificity 73.3%, and accuracy 73.6%). Overall, DSACR on POD3 had the highest AUC value. Furthermore, a multivariate logistic regression analysis revealed that pancreatic texture (soft; odds ratio [OR] 9.22; 95% confidence interval [CI] 2.22–44.19; *p* < 0.01) and DSACR on POD3 (> 22; OR 8.76; 95% CI 2.78–31.59; *p* < 0.001) were independently associated with POPF after DP.

**Conclusions:**

DSACR is the most reliable indicator of drain fluid amylase status for predicting POPF after DP.

## Background

Postoperative pancreatic fistula (POPF) is a major complication of pancreatic surgery. POPF causes secondary complications, such as abdominal abscess, delayed gastric emptying, and postoperative bleeding, and may result in a prolonged hospital stay and surgery-related death [1–3]. Although surgical procedures have been standardized and surgical devices have been developed in pancreatic surgery, the incidence of POPF has been reported to range from 3–50%, even at high-volume centers [4–7]. When limited to distal pancreatectomy (DP) cases, POPF still occurs at a rate of 24–39% [8–13].

Many studies have demonstrated the risk factors of POPF, such as pancreatic texture, body mass index (BMI), intraoperative blood loss, and postoperative drain fluid amylase concentration (DAC) [14–26]. Additionally, we have reported that DAC on postoperative day (POD) 3 after pancreatectomy can be a reliable indicator for predicting the development of POPF [27, 28]. However, there are other indicators related to the drain fluid amylase status such as daily drainage volume, drain fluid amylase amount (DAA), and drain fluid and serum amylase concentration ratio (DSACR). Few studies have compared these indicators [26, 29, 30]; therefore, the most reliable indicator for predicting POPF remains unclear. The object of this study is to identify the single most accurate indicator related to drain fluid amylase status of POPF after DP.

## Methods

### Patient

**Fig. 1:**
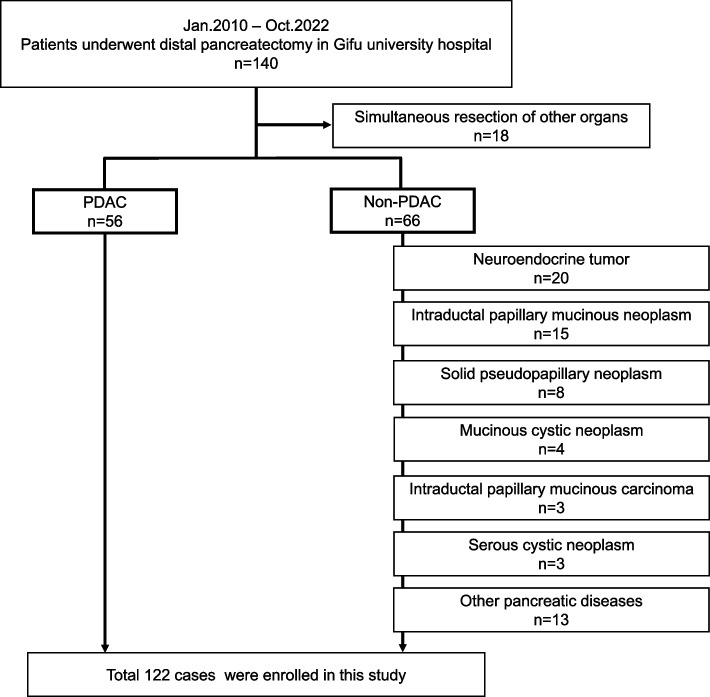
Exclusion criteria

This retrospective study included 140 patients who underwent DP for pancreatic disease at department of gastroenterological surgery in Gifu University Hospital between January 2010 and October 2022. All procedures were conducted by expert surgeons who had qualified through the board certification system of the Japanese Society of Hepato-Biliary-Pancreatic Surgery (JSHBPS). We excluded 18 patients (simultaneous resection of other organs); therefore, 122 patients were included in this study (Fig. 1). The study was conducted in accordance with the World Medical Association Declaration of Helsinki, and the Ethics Committee of Gifu University approved the study (approval number: 2022–157) [28].

### Perioperative management

In cases of DP for PDAC, regional lymph node dissection with splenectomy following the classification of pancreatic carcinoma by the Japan Pancreas Society [31] and pancreatic resection of the portal vein were performed. In the case of DP for non-PDAC, systematic lymph node dissection was omitted, and pancreatic resection was performed with a sufficient margin from the tumor using hand-sewn closure or a linear stapler.

In the hand-sewn closure group, the pancreas was resected after identifying the main pancreatic duct, which was ligated with a 3–0 silk suture. The stump of the remnant pancreas was closed with vertical mattress sutures using 5–0 polypropylene. In the linear stapler group, the pancreas was transected with a purple or black cartridge using the Endo GIA™ Tri-Staple or Signia™ stapling system (Medtronic plc., Dublin, Ireland). The closed jaw was clamped carefully and slowly for 5 min at a fixed speed. Firing was performed at a rate of 1 cm/min by firmly fixing the stapler. After firing, the jaws of the stapler were closed for 1 min. One 19-Fr. Blake silicon drain (Johnson & Johnson, Inc. New Brunswick, NJ, USA) was placed near the stump of the remnant pancreas. The drain was removed on POD4–5, when the drainage fluid was clear, postoperative course was stable, the patient had no abdominal pain, fever, or other symptoms, and no fluid collection in pancreatic stump on ultrasonography (US) or computed tomography (CT). The DAC and drainage volumes were measured from POD1 until the drain was removed. All patients received prophylactic antibiotics (cefmetazole) either intraoperatively or two days postoperatively.

### Definition of DAA and DSACR

DAA on POD1 was calculated by multiplying the DAC on POD1 and the 24-h drainage volume from the morning of POD1. DAA on POD3 was calculated in the same manner.

DSACR on POD1 was calculated by dividing the DAC on POD1 by serum amylase concentration (SAC) on POD1. DSACR on POD3 was calculated in the same manner.

### Definition of POPF

In this study, we included only clinically relevant POPF. Therefore, only Grades B and C pancreatic fistulas were defined as POPF (Grade B, symptomatic fistula requiring therapeutic intervention, such as antibiotics, drain placement for over 3 weeks, and percutaneous drainage; Grade C, symptomatic fistula associated with a severe general condition of patients, sepsis, and multi-organ failure requiring aggressive treatment in the intensive care unit with surgical intervention) based on the International Study Group of Pancreatic Fistula (ISGPF) definitions [32]. The day of POPF diagnosis was defined as intra-abdominal fluid collection with positive cultures identified by US or CT.

### Statistical analysis

Continuous and categorical variables are presented as median (range) values and frequencies (percentages), respectively. Fisher’s exact test was used for comparisons of variables between the POPF and non-POPF groups and for categorical variables. The Mann–Whitney U test was used in independent cases for comparisons of variables between groups, and the Wilcoxon signed-rank test was used in paired cases for continuous variables. The predictive ability of POPF after DP was assessed by calculating the area under the receiver operating characteristic (ROC) curve. Youden's index was used to determine the optimal cutoff value to calculate specificities and sensitivities in the ROC curve analysis. Variables identified as potentially significant by univariate analysis were selected for multivariate analysis using a logistic regression model to identify the independent predictors of POPF after DP. The limit of statistical significance for all analyses was defined as a two-sided p-value < 0.05. All statistical analyses were performed using JMP software (SAS Institute Inc., Cary, NC, USA).

## Results

### Comparison of patient characteristics and surgical outcomes between patients with and without POPF

Table 1 summarizes the characteristics of patients with and without POPF. POPF occurred in 30 (24.6%) patients. Among patients with POPF, Grade C POPF occurred in two patients (6.7%), and the remaining 28 patients (93.3%) had Grade B POPF. Age, sex, BMI, preoperative serum albumin level, history of diabetes mellitus, pancreatic disease, and tumor location did not significantly differ between the two groups.

**Table 1:** Comparison of characteristics between patients with and without POPF after distal pancreatectomy

	Patients with POPF (*n* = 30)	Patients without POPF (*n* = 92)	*p*-value
Age (years)	67 (40–82)	67 (11–84)	0.92
Sex			0.53
Male	19 (63.3)	52 (56.5)	
Female	11 (36.7)	40 (43.5)	
BMI (kg/m^2^)	23.6 (17.6–32.2)	22.6 (16.2–32.2)	0.12
Albumin (g/dl)	4.1 (3.3–5.0)	4.3 (2.7–4.9)	0.30
Diabetes mellitus	7 (23.3)	31 (33.7)	0.37
Pancreatic disease			0.53
PDAC	12 (40.0)	44 (47.8)	
Non-PDAC	18 (60.0)	48 (52.2)	
Location			0.40
Body	15 (50.0)	55 (59.8)	
Tail	15 (50.0)	37 (40.2)	

Data are expressed as median (range) or number of patients

*POPF* Postoperative pancreatic fistula, *BMI* Body mass index, *PDAC* Pancreatic ductal adenocarcinoma

^*^: *p* < 0.05

^**^: *p* < 0.01

^***^: *p* < 0.001

Table 2 shows the surgical outcomes of the two groups. No significant differences existed between the two groups in operative time, intraoperative blood loss, surgical procedures (open or laparoscopic surgery/spleen preservation or not), pancreatic stump closure method, and pancreatic thickness. A significant difference existed in the soft pancreatic texture rate (85.7% in the POPF group vs. 46.8% in the non-POPF group, *p* < 0.001). DAC, DAA, and DSACR on both POD1 and 3 were significantly higher in the POPF group than in the non-POPF group. However, postoperative SAC and daily drainage volume were not significantly different between the two groups.

**Table 2:** Comparison of surgical outcomes between patients with and without POPF after distal pancreatectomy

	Patients with POPF (*n* = 30)	Patients without POPF (*n* = 92)	*p*-value
Operative time (min)	285 (174–537)	270 (143–564)	0.12
Blood loss (ml)	190 (10–1910)	260 (0–1840)	0.65
Surgical procedure			
Open	24 (80.0)	62 (67.4)	0.19
Laparoscopic	6 (20.0)	30 (32.6)	
Spleen preserving	3 (10.0)	18 (19.6)	0.23
Non spleen preserving	27 (90.0)	74 (80.4)	
Stump closure method			0.81
Stapler	19 (63.3)	56 (60.9)	
Hand-sewn	11 (36.7)	36 (39.1)	
Pancreas texture			< 0.001^***^
Soft	24 (85.7)	37 (46.8)	
Hard	4 (14.3)	42 (53.2)	
Pancreas thickness (mm)	11 (8–17)	11 (3–24)	0.70
SAC (U/L)			
POD1	117 (47–1108)	158 (35–1921)	0.36
POD3	38 (11–223)	50 (15–663)	0.11
DAC (U/L)			
POD1	7977 (108–34,076)	1975 (42–61,075)	0.01^*^
POD3	1799 (42–16,515)	390 (35–43,873)	< 0.001^***^
Drainage volume (ml)^a^			
POD1	60 (12–355)	56 (2–475)	0.91
POD3	30 (5–205)	31 (3–540)	0.60
DAA (U/day)^b^			
POD1	235 (11–3748)	115 (1–3237)	0.02^*^
POD3	53 (2–1906)	16 (0.1–570)	< 0.001^***^
DSACR^c^			
POD1	34 (0.3–315)	14 (0.3–399)	< 0.01^**^
POD3	40 (1–403)	7 (0.7–593)	< 0.001^***^
Mortality	2 (6.9)	0 (0.0)	0.06
Postoperative hospital stays (days)	35 (12–121)	12 (7–23)	< 0.001^***^

Data are expressed as median (range) or number of patients

*POPF* Postoperative pancreatic fistula, *SAC* Serum amylase concentration (U/L), *DAC* Drain fluid amylase concentration (U/L), *DAA* Drain fluid amylase amount (U/day), *DSACR* Drain fluid and serum amylase concentration ratio, *POD* Postoperative day

^a^The 24 h drainage volume from the morning of POD1 and 3

^b^Calculated by multiplying drain fluid amylase concentration and drainage volume

^c^Calculated by dividing drain fluid amylase concentration by serum amylase concentration

^*^: *p* < 0.05

^**^: *p* < 0.01

^***^: *p* < 0.001

### Relationship between changes in DAC, DAA, and DSACR with and without POPF

**Fig. 2:**
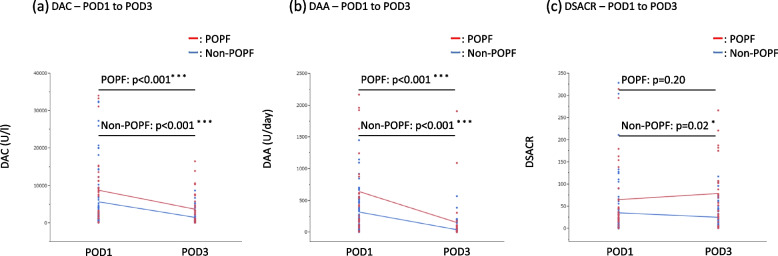
Relationship between changes in DAC, DAA, and DSACR with and without POPF. The Wilcoxon signed-rank test was conducted for comparisons between the groups. DAC, drain fluid amylase concentration; DAA, drain fluid amylase amount; DSACR, drain fluid and serum amylase concentration ratio; POPF, postoperative pancreatic fistula. *p < 0.05; ***p < 0.001

Both DAC and DAA significantly decreased from POD1 to 3, regardless of the presence or absence of POPF (all *p* < 0.001) (Fig. 2a and b). The DSACR also showed a significant decrease from POD1 to 3 in the non-POPF group (*p* = 0.02). Notably, no significant change was observed in DSACR in the POPF group (*p* = 0.20) (Fig. 2c).

### Cutoff values of DAC, DAA, and DSACR on POD1 and 3 for predicting POPF

**Fig. 3:**
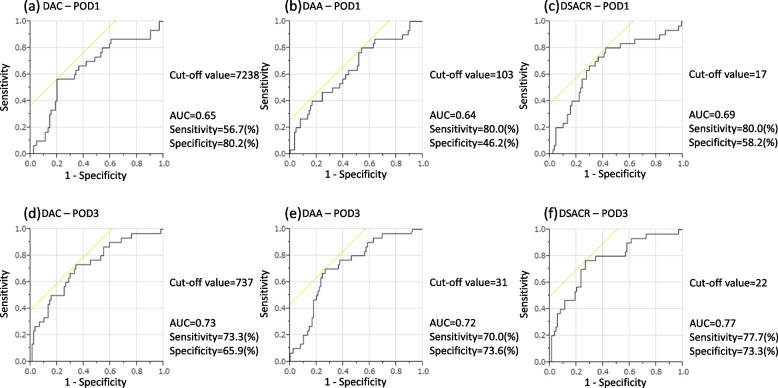
ROC curve analysis of DAC, DAA, and DSACR on postoperative days 1 and 3. ROC, receiver operating characteristics; DAC, drain fluid amylase concentration; DAA, drain fluid amylase amount; DSACR, drain fluid and serum amylase concentration ratio

Figure 3 and Table 3 show the ROC curves for generating the cutoff values of DAC, DAA, and DSACR on POD 1 and 3. The cutoff value of DAC on POD1 was 7238 (U/L), with an area under the curve (AUC) of 0.65, sensitivity of 56.7%, specificity of 80.2%, and accuracy of 74.4% (Fig. 3a). The cutoff value of DAA on POD1 was 103 (U/day), with an AUC of 0.64, sensitivity of 80.0%, specificity of 46.2%, and accuracy of 54.5% (Fig. 3b). The cutoff value of DSACR on POD1 was 17, with an AUC of 0.69, sensitivity of 80.0%, specificity of 58.2%, and accuracy of 63.6% (Fig. 3c). The cutoff value of DAC on POD3 was 737 (U/L), with an AUC of 0.73, sensitivity of 73.3%, specificity of 65.9%, and accuracy of 67.8% (Fig. 3d). The cutoff value of DAA on POD3 was 31 (U/day), with an AUC of 0.72, sensitivity of 70.0%, specificity of 73.6%, and accuracy of 72.7% (Fig. 3e). The cutoff value of the DSACR on POD3 was 22, with an AUC of 0.77, sensitivity of 77.7%, specificity of 73.3%, and accuracy of 73.6% (Fig. 3f). Generally, the results indicated that the most reliable indicator for predicting POPF after DP was the DSACR on POD3, which had the highest AUC value. Additionally, the AUC of all indicators tended to be higher on POD3 than on POD1.

**Table 3:** Accuracy of DAC/ DAA/ DSACR – POD1 and 3 to predict POPF after distal pancreatectomy

	Cutoff value	AUC	Sensitivity	Specificity	PPV	NPV	Accuracy
DAC – POD1	7238	0.65	56.7	80.2	48.6	84.9	74.4
DAC – POD3	737	0.73	73.3	65.9	41.5	88.2	67.8
DAA – POD1	103	0.64	80.0	46.2	32.9	87.5	54.5
DAA – POD3	31	0.72	70.0	73.6	46.7	88.2	72.7
DSACR – POD1	17	0.69	80.0	58.2	38.7	89.8	63.6
DSACR – POD3	22	0.77	77.7	73.3	48.9	90.4	73.6

*AUC* Area under the ROC curve, *PPV* Positive predictive value, *NPV* Negative predictive value, *POPF* Postoperative pancreatic fistula, *DAC* Drain fluid amylase concentration (U/L), *DAA* Drain fluid amylase amount (U/day), *DSACR* Drain fluid and serum amylase concentration ratio

### Uni- and multivariate analysis of prediction for POPF after DP

In the univariate logistic regression analysis, POPF after DP was significantly associated with pancreatic texture, and DSACR on POD1 and POD3 (all *p* < 0.001).

Multivariate logistic regression analysis revealed that pancreatic texture (soft: odds ratio [OR] 9.22; 95% confidence interval [CI] 2.22–44.19; *p* < 0.01) and DSACR on POD3 (> 22; OR 8.76; 95% CI 2.78–31.59; *p* < 0.0001) were independently associated with POPF after DP (Table 4).

**Table 4:** Uni- and multivariate predictive factors of POPF after DP

	n	Univariate			Multivariate		
		OR	95%CI	*p*-value	OR	95%CI	*p*-value
Age (years)							
> 70	49	0.82	0.34–1.90	0.65			
< 70	73	1					
Sex							
Male	71	1.32	0.58–3.18	0.51			
Female	51	1					
BMI (kg/m^2^)							
> 24	42	2.13	0.91–5.04	0.08	2.49	0.79–8.00	0.12
< 24	80	1			1		
Albumin (g/dl)							
> 3.6	112	0.45		0.26			
< 3.6	10	1					
Pancreatic disease							
PDAC	56	0.73	0.31–1.67	0.45			
Non-PDAC	66	1					
Location							
Body	70	0.67	0.29–1.55	0.35			
Tail	52	1					
Operative time (min)							
> 300	41	1.43	0.60–3.33	0.42			
< 300	80	1					
Blood loss (ml)							
> 400	42	0.92	0.37–2.17	0.85			
< 400	79	1					
Surgical procedure							
Open	86	1.93	0.75–5.67	0.18			
Laparoscopic	36	1					
Spleen preserving	20	0.46	0.10–1.49	0.21			
Non spleen preserving	102	1					
Resection procedure							
Hand-sewn	47	0.90	0.37–2.09	0.81			
Stapler	75	1					
Pancreas texture							
Soft	66	6.81	2.37–24.79	< 0.001^***^	9.22	2.22–44.19	< 0.01**
Hard	46	1			1		
Pancreas thickness (mm)			0.34–1.83	0.59			
> 12	58	0.80					
< 12	64	1					
SAC (U/L) –POD1							
> 100	88	0.58	0.71–4.16	0.22			
< 100	34	1					
SAC (U/L) –POD3							
> 100	16	1.01	0.27–3.20	0.98			
< 100	105	1					
Drainage volume (ml)^a^ – POD1							
> 40	89	1.66	0.64–4.89	0.31			
< 40	33	1					
Drainage volume (ml)^a^ – POD3							
> 55	35	0.70	0.25–1.74	0.45			
< 55	87	1					
DSACR^b^ – POD1							
> 17	63	5.33	2.09–15.54	< 0.001^***^	1.51	0.40–5.91	0.55
< 17	58	1			1		
DSACR^b^ – POD3							
> 22	47	9.04	3.59–25.33	< 0.001^***^	8.76	2.78–31.59	< 0.001***
< 22	73	1			1		

*POPF* Postoperative pancreatic fistula, *OR* Odds ratio, *95%CI* 95% confidence interval, *BMI* Body mass index, *PDAC* Pancreatic ductal adenocarcinoma, *SAC* Serum amylase concentration (U/L), *DSACR* Drain fluid and serum amylase concentration ratio, *POD* Postoperative day

^a^The 24 h drainage volume from the morning of POD1 and 3

^b^Calculated by dividing D-Amy concentration by S-Amy concentration

^*^: *p* < 0.05

^**^: *p* < 0.01

^***^: *p* < 0.001

## Discussion

Notwithstanding the attempts to reduce POPF frequency with the development of surgical techniques and devices, a high incidence of POPF is reported in pancreatic surgery [4–13].

Many studies have reported various predictive factors for POPF, including patient-related factors (e.g., sex and BMI), pancreas-related factors (e.g., pancreatic texture and thickness), and perioperative-related factors (e.g., stump closure method, intraoperative blood loss, and DAC) [14–28]. Particularly, many studies involved the association between postoperative drain amylase status and POPF [16–25]. We have reported that DAC on POD3 can significantly predict the development of POPF [27, 28]. However, while DAC is the most widely used drain amylase-related indicator, other indicators, such as DAA and DSACR, exist. The values of the DAA and DSACR consider the daily drainage volume and SAC, respectively, in the DAC. These indicators may be more reliable than DAC. We identified the DSACR on POD3 as the most reliable indicator for predicting POPF after DP.

The incidence of POPF varied significantly in different studies because of the definition of POPF used at each institution. Thus, in 2005, the ISGPF developed a definition and grading of POPF that has been universally accepted and unified [32]. Three basic parameters were considered in defining POPF for ISGPF: 1) amylase content in the drain fluid, 2) daily output volume of the drain fluid, and 3) duration. Finally, amylase content in drain fluid was defined as “greater than three times the serum amylase activity” and the duration was defined as “after postoperative day 3,” that is, “DSACR after POD3 > 3.” However, the daily output volume of the drain fluid was defined as “any measurable volume” and was excluded from the definition of POPF.

Once POPF develops, enzymatic fluid leaks into the abdominal cavity from the pancreatic stump, resulting in an increased amylase content in the drain fluid. Therefore, prior to this study, we hypothesized that DAA is a more reliable predictor than DAC because drainage quantity is being considered. However, the study results revealed that DAA was the least reliable of the three indicators related to drain amylase status as a predictor of POPF after DP. Previous studies have focused on postoperative drainage volume and the total amount of amylase in the drainage fluid [22, 26, 29, 30]; however, the results are not always consistent. Molinari et al. [22] reported no significant difference in drainage volume between patients with and without POPF, similar to our results. Conversely, Fukami et al. [26] reported that the median drainage volumes on POD1 and 3 were significantly lower in the POPF group than in the non-POPF group. This result could be due to the reduced drainage efficiency by the highly turbid drain fluid and the dense adhesion around the pancreatic stump in the POPF group. We suspected that the use of a non-suctioned (gravity) drain in their study, unlike in our study, may have been a reason for the difference in results. However, their conclusion that DAA was inferior to DAC as a predictor of POPF was consistent with our findings. Sakamoto et al. [29] reported an analysis of POPF prediction limited to DP cases, similar to this study. They reported that the DAC value on both POD1 and 3 did not have a significant correlation with POPF, whereas DAA had a significant correlation. Okano et al. [30] investigated the predictive ability of DAA in 54 patients who underwent pancreatectomy, and they reported that neither DAC nor DAA were significant predictors of POPF, whereas the persistence ratio of DAA between POD1 and 3 was significant. Although further investigation is required, it would be difficult to make DAA a universal indicator, as it may be affected by the properties of the drain fluid, type of drain, and condition of the pancreatic stump.

While DAC is undoubtedly an excellent predictor of POPF, it is difficult to set appropriate cutoff values. This is because pancreatic exocrine function varies from case to case, and thus the threshold for predicting POPF is likely to be different in each case. Another reason is that DAC fluctuates significantly over time after surgery. This study showed a significant decrease from POD1 to 3 with and without POPF. Consequently, the cutoff value for predicting POPF in previous studies varied from 1300 to 5000 for POD1 and 737 to 3000 for POD3 [16–28]. Therefore, it is presumed that the accuracy of DAC would be further improved if it reflects the pancreatic exocrine function of individual cases and is less prone to change with time after surgery. In this study, we observed that only the DSACR showed no significant changes from POD1 to 3 in the POPF group. Furthermore, the cutoff values of DSACR for predicting POPF were only slightly different between POD1 and 3 (optimal cutoff values of DSACR: 17 and 22, respectively), unlike other indicators (DAC: 7238 and 737 U/l, DAA: 103 and 31 U/day, respectively). To the best of our knowledge, few studies have evaluated the usefulness of DSACR for predicting POPF during the postoperative period. Based on the results of this study, we propose that the most reliable predictive indicator for POPF after DP is DSACR on POD3. DSACR may contribute to both improving diagnostic accuracy and resolving difficulties in setting optimal cutoff values.

This study had some limitations. First, it was single-center retrospective study with a small sample size. This sample size may have caused selection bias and multiplicity issues in statistical analysis. This limitation should be considered when evaluating the results of this study. A prospective multicenter study with a larger number of patients is required for more accurate results. Second, technical variations existed in the DP surgical procedure, such as open or laparoscopic, spleen-preserving or non-preserving, pancreas transection methods, and with and without lymph node dissection. This study showed no significant correlation between these surgery-related factors and POPF. It is expedient to unify surgical procedures to calculate more appropriate cutoff values for DSACR.

## Conclusions

We discovered DSACR to be the most reliable indicator for predicting POPF by comparing several indicators related to the drain fluid amylase status.

## Data Availability

The datasets used during the current study are available from the corresponding author on request.

## Abbreviations

POPF: Postoperative pancreatic fistula
DP: Distal pancreatectomy
BMI: Body mass index
DAC: Drainage fluid amylase concentration
POD: Postoperative day
DAA: Drain fluid amylase amount
DSACR: Drainage fluid and serum amylase concentration ratio
JSHBPS: Japanese Society of Hepato-Biliary-Pancreatic Surgery
SAC: Serum amylase concentration
ISGPF: International Study Group of Pancreatic Fistula
US: Ultrasonography
CT: Computed tomography
ROC: Receiver operating characteristic
AUC: Area under the curve
OR: Odds ratio
CI: Confidence interval
